# Genetic variation within the pri-let-7f-2 in the X chromosome predicting stroke risk in a Chinese Han population from Liaoning, China: From a case-control study to a new predictive nomogram

**DOI:** 10.3389/fmed.2022.936249

**Published:** 2022-11-30

**Authors:** Yaxuan Wang, Luying Qiu, Yuye Wang, Zhiyi He, Xue Lan, Lei Cui, Yanzhe Wang

**Affiliations:** ^1^Department of Anesthesiology, The First Affiliated Hospital of China Medical University, Shenyang, China; ^2^Department of Neurology, Key Laboratory for Neurological Big Data of Liaoning Province, The First Affiliated Hospital of China Medical University, Shenyang, China; ^3^School of Health Management, China Medical University, Shenyang, China

**Keywords:** ischemic stroke, pri-microRNA, pri-let-7f-2, single nucleotide polymorphism (SNP), rs17276588, case-control study, predictors, nomogram

## Abstract

**Background and objectives:**

Stroke is the most common cause of disability and the second cause of death worldwide. Therefore, there is a need to identify patients at risk of developing stroke. This case-control study aimed to create and verify a gender-specific genetic signature-based nomogram to facilitate the prediction of ischemic stroke (IS) risk using only easily available clinical variables.

**Materials and methods:**

A total of 1,803 IS patients and 1,456 healthy controls from the Liaoning province in China (Han population) were included which randomly divided into training cohort (70%) and validation cohort (30%) using the sample function in R software. The distribution of the pri-let-7f-2 rs17276588 variant genotype was analyzed. Following genotyping analysis, statistical analysis was used to identify relevant features. The features identified from the multivariate logistic regression, the least absolute shrinkage and selection operator (LASSO) regression, and univariate regression were used to create a multivariate prediction nomogram model. A calibration curve was used to determine the discrimination accuracy of the model in the training and validation cohorts. External validity was also performed.

**Results:**

The genotyping analysis identified the A allele as a potential risk factor for IS in both men and women. The nomogram identified the rs17276588 variant genotype and several clinical parameters, including age, diabetes mellitus, body mass index (BMI), hypertension, history of alcohol use, history of smoking, and hyperlipidemia as risk factors for developing IS. The calibration curves for the male and female models showed good consistency and applicability.

**Conclusion:**

The pri-let-7f-2 rs17276588 variant genotype is highly linked to the incidence of IS in the northern Chinese Han population. The nomogram we devised, which combines genetic fingerprints and clinical data, has a lot of promise for predicting the risk of IS within the Chinese Han population.

## Introduction

Stroke is the most common cause of disability and the second cause of death worldwide (1, 2). However, in China, stroke is the leading cause of disability and death (3, 4). The most common type of stroke is ischemic stroke (IS) which accounts for 70% of all cases (5). The long-term disability caused by stroke has a substantial socioeconomic impact (6). As a result, there is a need to develop reliable IS risk prediction tools that could be used to monitor high-risk patients to reduce the incidence of IS and improve treatment outcomes.

Since the traditional IS risk factors, such as age, gender, hypertension, and diabetes mellitus, other fail to predict the disease adequately, various previous studies have evaluated the role of genetic factors in predicting the disease (7). For example, some studies have shown that the single nucleotide polymorphism (SNP) within the primary (pri-), precursor, and mature micro-ribonucleic acid (miRNAs) can influence the target selection or the expression level of miRNAs leading to an increased risk of developing IS (8–11). Let-7f, a member of the let-7 family, is a highly conserved within the human miRNA in sequence and function (12). The let-7f genes are involved in several biological processes, such as atherosclerosis, angiogenesis, neuroinflammation, and endothelial function, which play important roles in the pathological process of IS. Furthermore, the let-7f antagonist was associated with neuroprotective properties in IS (13). In particular, the pri-let-7f-2 rs17276588 polymorphism on the X-chromosome was found to increase the risk of developing several diseases, including colorectal cancer and metabolic syndrome (MetS), and may also have a role in the development of IS (14, 15). However, the relationship between the pri-let-7f-2 rs17276588 polymorphism and IS risk has not been reported. Therefore, there is a need to identify whether this gene could be used as a novel predictive target for IS in combination with other established clinical risk factors.

Nomograms are statistical techniques that are often used to develop risk prediction models for the development of specific diseases and can be applied to quantify an individual’s risk of developing IS (16–18). Therefore, this study aimed to perform a case-control study to evaluate the role of the pri-let-7f-2 rs17276588 polymorphism in predicting IS in both males and females. This genomic model was combined with other known clinical risk factors to develop a gender-specific, genetic signature-based nomogram for predicting IS.

## Materials and methods

### Data collection

**FIGURE 1:**
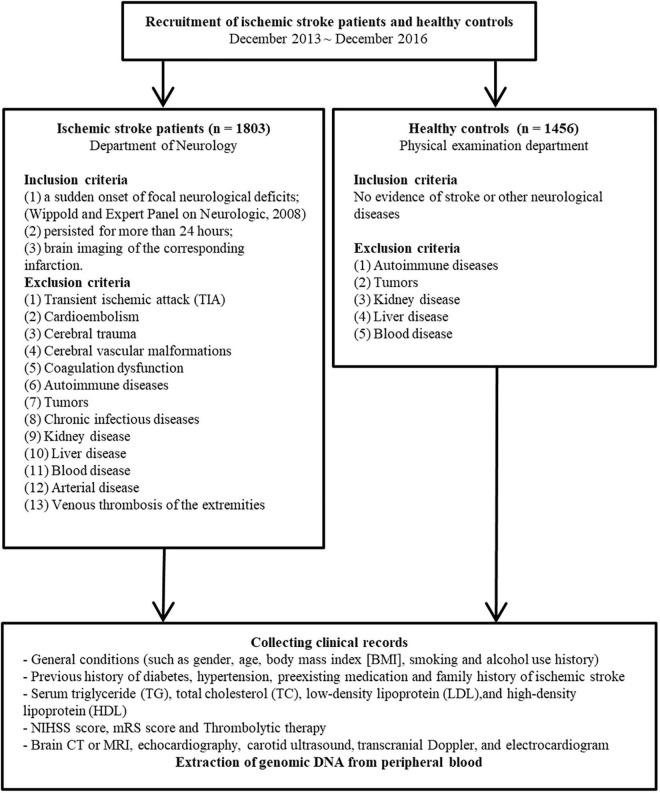
Patient and controls enrollment flowchart. (NIHSS, National Institutes of Health Stroke Scale; mRS, modified Rankin Scale).

There were 1,858 men and 1,401 women in our age-matched sample. From December 2013 to December 2016, patient data were collected at the First Affiliated Hospital of China Medical University. The eligibility criteria for both patients and controls were defined as per our previously published studies (8–10) and are illustrated in Figure 1.

### Ethical considerations

Written informed consent was obtained from all patients enrolled in this study. This study was approved by the Institutional Ethical Committee of the First Affiliated Hospital of China Medical University on February 20, 2012 (No. 2012-38-1), and it was carried out in accordance with the declaration of Helsinki. The protocol was submitted to the Chinese Clinical Trial Registry (registration number: ChiCTR-COC-17013559).

### Single nucleotide polymorphism selection and genotyping

The University of California, Santa Cruz (UCSC) genome browser^1^ and the SNP database (dbSNP)^2^ were used to find the relevant SNPs. The SNPs were included if they were situated −1 kb upstream of the pri-let-7f-2 on the X chromosome, had a minor allele frequency above 0.1 in the Han Chinese population, and were identified as possible functional SNPs by the National Institute of Environmental Health Sciences’ SNP function prediction (FuncPred) program.^3^ One relevant SNP, rs17276588, was discovered at −183 bp upstream of the pri-let-7f-2 on the X chromosome (Table 1).

**TABLE 1:** Characteristics of the rs17276588 single nucleotide polymorphism (SNP) selected for the study.

SNP ID	Gene	Chr	Genomic location	Genic position	Alleles (major/minor)	MAF
rs17276588	pri-let-7f-2	x	53601143	5-near	G/A	0.27

MAF, minor allele frequency.

The DNA purification kit Promega (Madison, WI, USA), was used to extract genomic DNA from the peripheral blood, and the SNaPshot reaction was done as previously described (8–10). The genotyping analysis was done using the SNaPshot Multiplex kit (Applied Biosystems Co., Ltd., Foster City, CA, USA). The ABI 3130XL DNA sequence detector and GeneMapper 4.0 were used to analyze the data (Applied Biosystems Co., Ltd.).

### Statistical analyses

Computer-generated random numbers were used to assign patients to the training or validation cohorts in a 7:3 ratio. Variations in the distribution of the demographic variables, risk factors, and allele genotypes between the patient and control groups were assessed using the Pearson’s chi-square (χ^2^) test. The Hardy-Weinberg equilibrium (HWE) was tested for each genotype using the goodness-of-fit χ^2^-test. The frequency distribution of the categorized parameters was compared between the training and validation cohorts using the χ^2^ test or the Fisher exact test. The optimal predictive clinical and genomic risk variables for IS were identified using the least absolute shrinkage and selection operator (LASSO) method (19, 20). The odds ratios (OR) at a 95 percent confidence interval (CI) for each of the IS risk features were calculated using univariate and multivariate logistic regression methods.

The sociodemographic variables were included in the nomogram if the multivariate logistic regression model resulted in a *p*-value smaller than 0.05. The clinical features were combined with the risk factors identified using the LASSO regression, univariate and multivariate logistic regression analyses, and incorporated into the nomogram (21–23). Points were assigned to the nomogram by drawing a vertical line from each predictor-associated value to the axis points. The total sum of the assigned points for each predictor was calculated. All potential predictors were utilized to construct the predictive IS risk model.

The discrimination and calibration of the nomogram were assessed using Harrell’s concordance index (C-index) and calibration curves. The C-index was corrected using bootstrapping validation (1,000 bootstraps resamples) (24). In addition, the optimal cutoff point of the area under the curve (AUC) of a receiver operator characteristics (ROC) curve was also calculated. A calibration plot with 1,000 bootstraps resamples was used to test the calibration of the model.

A decision curve analysis was used to measure the net benefits at different threshold probabilities in the IS cohort (25). The net benefit was estimated by removing all false positive predictions and by evaluating the repercussions of avoiding interventions versus the negative effects of an unnecessary intervention (26). The nomogram was externally validated (27).

All statistical tests were carried out using the R statistical software (Version 3.1.1^4^), and a two-sided *p*-value below 0.05 was deemed statistically significant.

## Results

### Basic characteristics of the study subjects

A total of 1,858 male (training cohort: 1,394 and validation cohort: 464) and 1,401 female (training cohort: 1,051 and validation cohort: 350) participants were included in the study. The fundamental characteristics of the IS patients and controls, as well as IS risk factors, are outlined. The findings of the univariate analysis are summarized in Supplementary Tables 1A,B. The univariate analysis revealed no significant difference between the training and validation cohorts (Table 2 and Supplementary Tables 2A,B).

**TABLE 2:** Clinical and demographic characteristics of the patients in the training and validation cohorts.

Characteristics	Training cohort *(n* = *2445)*	Validation cohort *(n* = *814)*	*P*-value
Male, *n* (%)	1,394 (57.0)	464 (57.0)	0.984
Age (≤60/>60)	933 (38.2)	322 (39.6)	0.121
BMI (≤22.9/>22.9)	1,093 (44.7)	383 (47.1)	0.261
Diabetes mellitus, *n* (%)	584 (23.9)	185 (22.7)	0.531
Hypertension, *n* (%)	1,308 (53.5)	451 (55.4)	0.344
History of smoking, *n* (%)	858 (35.1)	317 (38.9)	0.052
History of alcohol use, *n* (%)	412 (16.9)	158 (19.4)	0.107
Hyperlipidemia, *n* (%)	1,082 (44.3)	324 (39.8)	0.053
	HW pval	HW pval	HW pval
rs17276588 G→A	*(n* = *2445)*	*(n* = *814)*	*(n* = *3259)*
	0.99	0.8571	0.95

BMI, body mass index.

### Association between the pri-let-7f-2 polymorphism and ischemic stroke

Since the locus of the pri-let-7f-2 gene is on the X chromosome, the allele and genotype frequencies of the rs17276588 polymorphisms were studied in both sexes separately, as shown in Table 3. The findings of the genotype analysis in the male population revealed that the A allele significantly increased the risk of developing IS when compared with the G allele (OR = 4.454, CI = 3.536–6.126, *p* < 0.001). In the female population, both the AA genotype (OR = 5.205, CI = 2.669–10.153, *p* < 0.001) and GA genotype (OR = 4.542, CI = 3.410–6.065, *p* < 0.001) significantly increased the risk of developing IS when compared with the reference GG genotype. The A allele significantly increased the risk of developing IS in both dominant (OR = 4.618, CI = 3.498–6.095, *p* < 0.001) and recessive (OR = 2.965, CI = 1.533–5.733, *p* = 0.001) forms.

**TABLE 3:** Allele and genotype frequencies of rs17276588 polymorphism among ischemic stroke (IS) cases and controls their impact on the risk of developing ischemic stroke (IS).

SNP	Cases	Percent (%)	Controls	Percent (%)	OR (95% CI)*^a^	*P-*value^b^
**Males**						
G(ref)	391	51.4	540	85.2	ref (–)	
A	369	48.6	94	14.8	4.654 (3.536–6.126)	< 0.001
**Females**						
GG(ref)	249	42.7	365	78	ref (–)	
GA	288	49.4	91	19.4	4.542 (3.410–6.065)	< 0.001
AA	46	7.9	12	2.6	5.205 (2.669–10.153)	< 0.001
Dominant model GA + AA vs. GG					4.618 (3.498–6.095)	< 0.001
Recessive model AA vs. GA + GG					2.965 (1.533–5.733)	0.001

SNP, single nucleotide polymorphism; OR, odds ratio; CI, confidence interval. *ORs and 95% CIs were calculated by logistic regression. ^a,b^Adjusted OR (95%CI) and *P*-value, adjusted for age, BMI, diabetes mellitus, hypertension, history of smoking, history of alcohol use and hyperlipidemia (BMI, body mass index).

### Predicted second structure of the pri-let-7f-2 mRNAs

**FIGURE 2:**
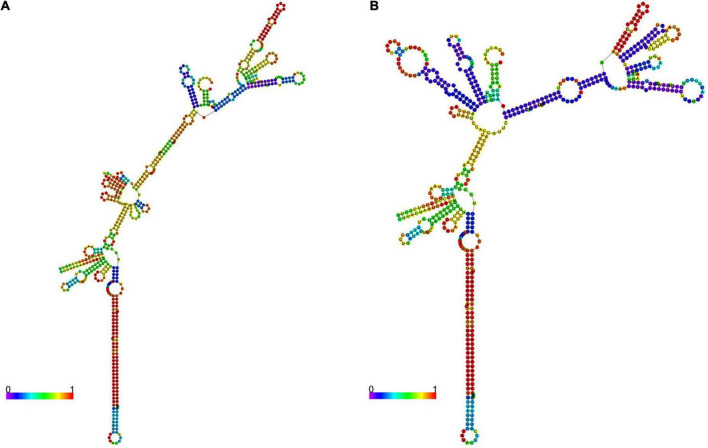
The pri-let-7f-2 mRNA secondary structures. Two 601-nt long pri-let-7f-2 DNA sequences centered on the rs17276588 polymorphism were inserted into the rs17276588- G **(A)** and rs17276588- A allele **(B)** RNA fold. The minimum free values (MAF) and figures were obtained from the RNA fold online tool (http://rna.tbi.univie.ac.at).

The RNA fold online tool^5^ was used to predict the minimum free energy (MFE) required to change the pri-let-7f-2 rs17276588 mutation to the local secondary structure of the pri-let-7f-2 mRNA. When the nucleotide at the pri-let-7f-2 rs17276588 locus changed from G to A, the MFE changed from −172.7 kcal/mol to −172.6 kcal/mol (Figure 2).

### Development of an individualized prediction model

**FIGURE 3:**
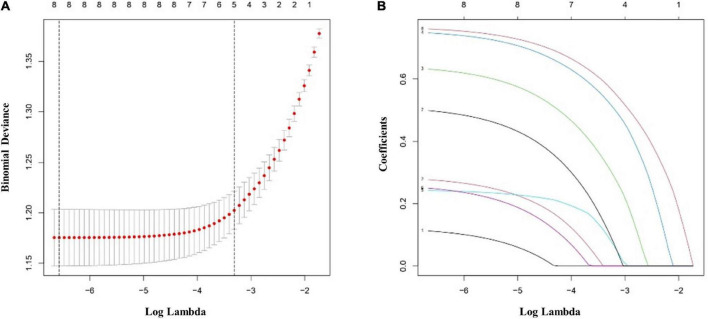
Clinical and demographic ischemic stroke (IS) risk factors in the male population according to the least absolute shrinkage and selection operator (LASSO) binary logistic regression model. **(A)** Tenfold cross-validation based on the minimum criterion was used to find the optimal parameter (lambda) selection for the LASSO model. A plot of the partial likelihood deviance (binomial deviance) curve versus log(lambda) is shown. The dashed vertical lines were plotted based on the optimal minimum criteria and 1-SE of the minimum criteria (1-SE criteria). **(B)** The eight feature LASSO coefficient profiles. A coefficient profiler was generated based on the log(lambda) sequence. A vertical line was plotted using the value chosen by the 10-fold cross-validation. The optimal lambda was defined as the value that yielded non-zero coefficients for all eight features. (SE, standard error; LASSO, least absolute shrinkage and selection operator).

**FIGURE 4:**
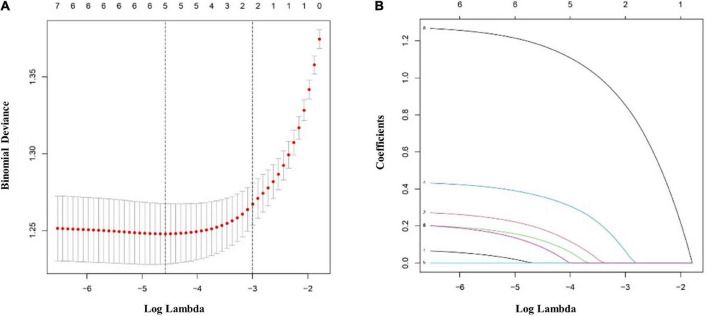
Clinical and demographic ischemic stroke (IS) risk factors in the female population according to the least absolute shrinkage and selection operator (LASSO) binary logistic regression model. **(A)** Ten-fold cross-validation based on the minimum criterion was used to find the optimal parameter (lambda) selection for the LASSO model. A plot of the partial likelihood deviance (binomial deviance) curve versus log(lambda) is shown. The dashed vertical lines were plotted based on the optimal minimum criteria and 1-SE of the minimum criteria (1-SE criteria). **(B)** The eight feature LASSO coefficient profiles. A coefficient profiler was generated based on the log(lambda) sequence. A vertical line was plotted using the value chosen by the tenfold cross-validation. The optimal lambda was defined as the value that yielded non-zero coefficients for all eight features. (SE, standard error; LASSO, least absolute shrinkage and selection operator).

The multivariate logistic regression findings for the IS risk factors are summarized in Tables 4A,B. In the male population, a body mass index (BMI) above 22.9 kg/m^2^ [OR, 1.335 (95% CI, 1.050–1.699); *p* = 0.018], diabetes mellitus [OR, 1.907 (95% CI, 1.425–2.563); *p* < 0.001], hypertension [OR, 2.134 (95% CI, 1.668–2.733); *p* < 0.001], hyperlipemia [OR, 1.674 (95% CI, 1.319–2.128); *p* < 0.001], and rs17276588 [AA versus GG; OR, 4.654 (95% CI, 3.548–6.150); *p* < 0.001] were identified as independent predictors for IS (Table 4A). In the female population, a BMI above 22.9 kg/m^2^ [OR, 1.334 (95% CI, 1.022–1.742); *p* = 0.034], hypertension [OR, 1.549 (95% CI, 1.177–2.039); *p* = 0.002], and rs17276588 [GA versus AA; OR, 4.542 (95% CI, 3.413–6.088); *p* < 0.001 and GG versus AA; OR, 5.205 (95% CI, 2.751–10.585); *p* < 0.001] were identified as independent predictors for IS (Table 4B). The LASSO analysis and cross-validation results illustrated that the optimal lambda (λ) value was 0.00140712 for the 1,394 male participants in the training cohort. The eight clinical risk factors screened in the male training cohort (Figures 3A,B) were identified as the best risk factors in the LASSO regression model (Supplementary Table 3). On the other hand, five clinical features, including high BMI, hypertension, history of alcohol use, diabetes mellitus and rs17276588 had non-zero coefficients in the female training cohort (λ = 0.01026424) (Supplementary Table 3 and Figures 4A,B).

**TABLE 4A:** Multivariate logistic regression analysis for the ischemic stroke (IS) risk factors among the male population.

Intercept and variables	Prediction model		
	β	OR (95% CI)	*P-*value
Intercept	–1.370	0.254 (0.194–0.331)	<0.001
Age (≤60/>60)	0.125	1.134 (0.884–1.455)	0.324
BMI (≤22.9/>22.9)	0.289	1.335 (1.050–1.699)	0.018
Diabetes mellitus	0.646	1.907 (1.425–2.563)	<0.001
Hypertension	0.758	2.134 (1.668–2.733)	<0.001
History of smoking	0.246	1.279 (0.953–1.716)	0.101
History of alcohol use	0.265	1.303 (0.910–1.871)	0.149
Hyperlipidemia	0.515	1.674 (1.319–2.128)	<0.001
rs17276588 AA vs. GG	1.538	4.654 (3.548–6.150)	<0.001

β is the regression coefficient. BMI, body mass index.

**TABLE 4B:** Multivariate logistic regression analysis for the ischemic stroke (IS) risk factors among the male population.

Intercept and variables	Prediction model		
	β	OR (95% CI)	*P-*value
Intercept	–0.873	0.418 (0.309–0.561)	<0.001
Age (≤60/>60)	0.095	1.100 (0.837–1.444)	0.495
BMI (≤22.9/>22.9)	0.288	1.334 (1.022–1.742)	0.034
Diabetes mellitus	0.224	1.251 (0.907–1.729)	0.174
Hypertension	0.437	1.549 (1.177–2.039)	0.002
History of smoking	0.031	1.032 (0.754–1.411)	0.846
History of alcohol use	0.200	1.221 (0.841–1.777)	0.295
Hyperlipidemia	0.022	1.022 (0.777–1.345)	0.874
rs17276588 AG vs. GG	1.513	4.542 (3.413–6.088)	<0.001
rs17276588 AA vs. GG	1.650	5.205 (2.751–10.585)	<0.001

β is the regression coefficient. BMI, body mass index.

### Apparent performance of the risk nomogram in the cohort

**FIGURE 5:**
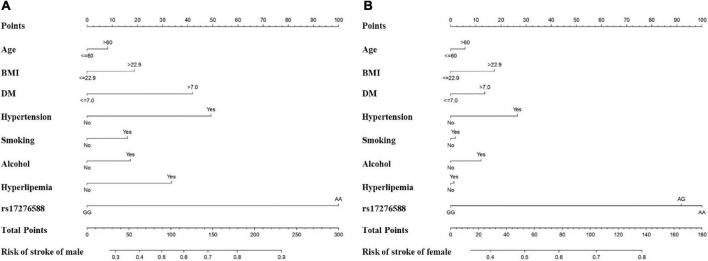
Nomogram for predicting the incidence of IS among the male **(A)** female **(B)** populations. The features included in the nomogram were age, BMI, DM, hypertension, smoking, alcohol, hyperlipemia, and rs17276588. (DM, Diabetes mellitus; BMI, body mass index).

**FIGURE 6:**
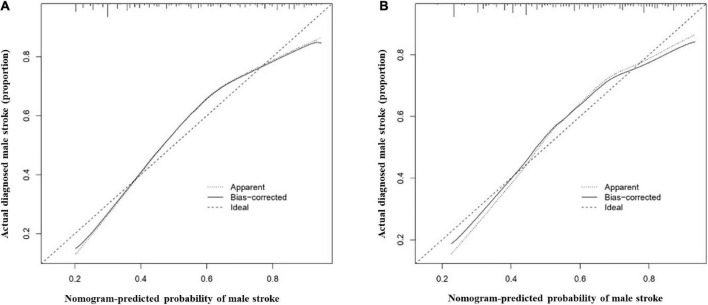
Calibration curves for the male nomogram prediction models. **(A)** illustrates the nomogram for the training cohort (*n* = 1394) with a mean absolute error of 0.035 and **(B)** illustrates the nomogram for the validation cohort (*n* = 464) with a mean absolute error of 0.030. The *x*-axis indicates the predicted IS incidence and the *y*-axis indicates the actual incidence of IS. The dotted diagonal line shows the model for perfect prediction, while the solid line illustrates the performance of the nomogram. The shorter the distance between the dashed line and the diagonal line, the higher the prediction accuracy of the model.

**FIGURE 7:**
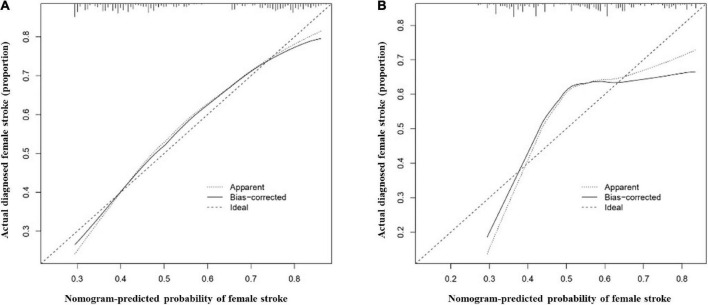
Calibration curves for the female nomogram prediction models. **(A)** illustrates the nomogram for the training cohort (*n* = 1051) with a mean absolute error of 0.017 and **(B)** illustrates the nomogram for the validation cohort (*n* = 350) with a mean absolute error of 0.062. The *x*-axis indicates the predicted IS incidence and the *y*-axis indicates the actual incidence of IS. The dotted diagonal line shows the model for perfect prediction, while the solid line illustrates the performance of the nomogram. The shorter the distance between the dashed line and the diagonal line, the higher the prediction accuracy of the model.

The most important predictors were used to create the nomogram (Figures 5A,B). In the training cohort, the C-index of the nomogram in male population was 0.767 (95% CI, 0.741–0.792), and in the validation cohort, it was 0.749 (95% CI, 0.703–0.795). The C-index of the nomogram in female population was 0.721 (95% CI, 0.690–0.752) in the training cohort and 0.684 (95% CI, 0.628–0.740) in the validation cohort. Both male and female calibration curves demonstrated good consistency and practicability. The mean absolute calibration error for the male population was 0.035 in the training cohort (Figure 6A) and 0.030 in the validation cohort (Figure 6B). The mean absolute calibration curve error for the female population was 0.017 in the training cohort (Figure 7A) and 0.062 in the validation cohort (Figure 7B).

### Clinical practicability

**FIGURE 8:**
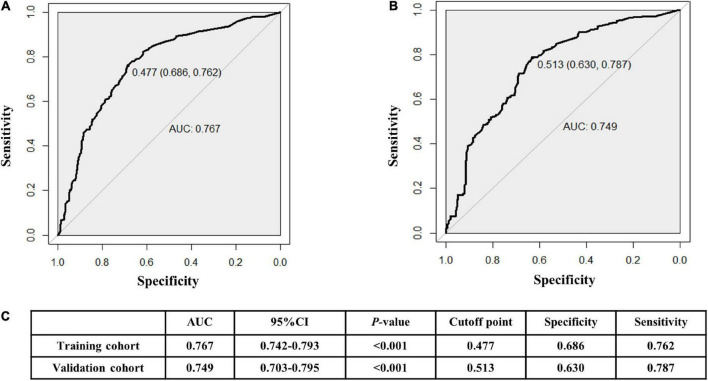
Receiver operating characteristic (ROC) curves for the male training **(A)** and validation **(B)** cohorts. **(C)** illustrates some of the results of the ROC curve for the training and validation nomograms.

**FIGURE 9:**
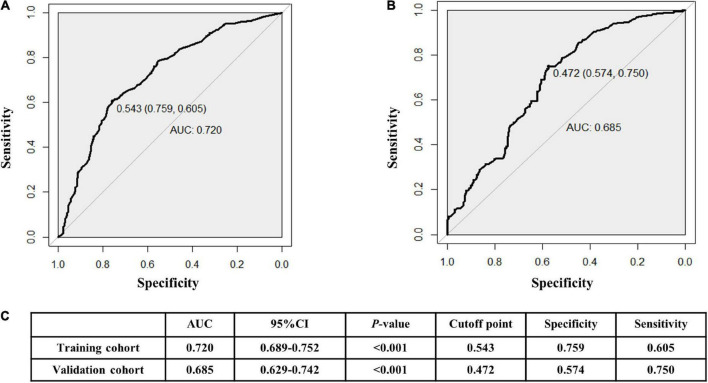
Receiver operating characteristic (ROC) curves for the female training **(A)** and validation **(B)** cohorts. **(C)** illustrates some of the results of the ROC curve for the training and validation nomograms.

**FIGURE 10:**
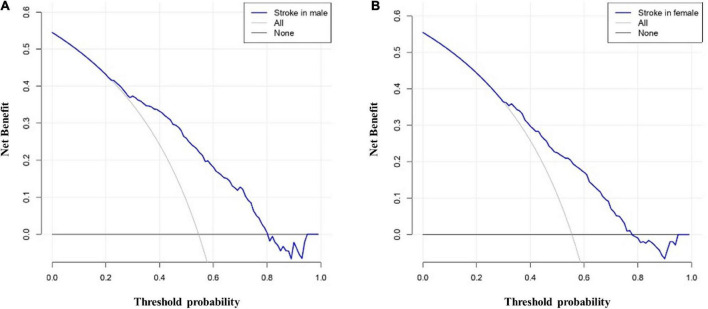
Decision curve analysis whereby the *y*-axis represents the net benefit, and the blue line represents the risk. The thin solid line denotes the assumption that all patients had IS, while the thick solid line denotes the assumption that no patients had IS. **(A)** illustrates the decision curve analysis for the male patients with the highest net benefit achieved for a threshold probability ranging between 21 and 80%. **(B)** illustrates the decision curve analysis for the female patients with the highest net benefit achieved for a threshold probability ranging between 31 and 77%. These findings indicate that an intervention based on the risk predicted by the nomogram provided a favorable net benefit when compared to treating all patients or treating none of the patients.

The AUCs in the training and validation male cohorts were 0.767 (95% CI, 0.742–0.793; *p* < 0.001) (Figures 8A,C) and 0.749 (95% CI, 0.703–0.795; *p* < 0.001) (Figures 8B,C), respectively. Similarly, the AUCs in the training and validation female cohorts were 0.720 (95% CI, 0.689–0.752; *p* < 0.001) (Figures 9A,C) and 0.685 (95% CI, 0.629–0.742; *p* < 0.001) (Figures 9B,C). These findings indicate that the nomograms for both male and female had a good prediction accuracy. Figure 10 shows the decision curve analysis for the nomogram, which was used to assess the clinical practicability of the model. The risk threshold whereby the patients would benefit clinically ranged between 21 and 80% for the male population (Figure 10A) and between 31 to 77% for the female population (Figure 10B).

## Discussion

In order to improve the primary prevention of IS, there is a need to develop accurate predictive models. However, current predictive models do not incorporate genetic risk factors such as the pri-let-7f-2 polymorphism on the X chromosome. In this study, we created and validated the first genotype-based nomogram for sex-specific IS prediction. The nomogram included two types of parameters, the rs17276588 genotype and clinical risk factors (age, diabetes mellitus, BMI, hypertension, history of alcohol use, history of smoking, and hyperlipidemia). According to the decision curve analysis, the nomogram proved clinically practical for both men and women.

Our findings indicate that the rs17276588 polymorphism is likely to be linked to an increased risk of developing IS. Since the A allele could be a risk factor for IS, individuals with the AA genotype may have a higher risk of developing IS. Studies have linked the let-7f genes with tumor cell growth, migration, and differentiation (28, 29). Therefore, previous studies focused on the role of the rs17276588 polymorphism in the development of various cancers (30). A study by Yuan et al. noted that when the rs17276588 A allele transcription was lower, the expression of the let-7f genes were reduced, resulting in a higher risk of developing colorectal cancer (14). Qian et al. evaluated the impact of the let-7f genes on the endothelial function of young stroke patients (31). These genes were identified as potential biomarkers to diagnose IS and predict prognosis in IS patients. In addition, a link was discovered between the rs17276588 variant genotypes GA and AA and an increased risk of MetS (15). This disease is characterized by an aberrant metabolic system that leads to various clinical problems such as diabetes and dyslipidemia (32). The metabolic impact of the rs17276588 SNPs suggests a possible relationship between SNP and IS.

Therefore, in this study, we used seven readily risk factors and the A allele in the pri-let-7f-2 rs17276588 to develop and validate a novel IS risk prediction tool. Our model accurately predicted the risk of IS for both male and female patients. Furthermore, the large sample size used to develop the model and the high C-index, calibration, and discrimination achieved following external validation indicate that the nomogram is generalizable and reliable. The 10-fold LASSO cross-validation identified high BMI, hypertension, history of alcohol use, diabetes mellitus and the rs17276588 A allele as high-risk factors for the development of IS. Conversely, age above 60, smoking history, and hyperlipidemia were not identified as IS risk factors in females. Hyperlipidemia was only identified as a risk factor in men following a multivariate logistic regression analysis in men. Our findings indicate that the male rs17276588 A allele carriers with hyperlipidemia had a higher IS risk than female patients. In previous studies, male sex and hyperlipidemia were also identified as independent risk factors for coronary heart disease and IS (33, 34). The triglyceride-glucose index (TyG) was also higher in men which was increased with age and serum total cholesterol (TC), low density lipoprotein cholesterol (LDL-C), triglyceride (TG) levels. In addition, patients with DM and hyperlipidemia had higher TyG and a higher risk of developing cardiovascular disease (CVD) (35–37). These findings suggest that a low-sodium and low-fat diet should be recommended, particularly for males with specific genotypes, to reduce the risk of developing IS. The clinical factors used in our nomogram can be easily obtained using routine tests, and it can therefore be easily applied in routine clinical practice, particularly to assess the IS risk in patients with the pri-let-7f-2 rs17276588 polymorphism. We also recommend regular check-ups and a low-fat diet in high-risk carriers to reduce the risk of developing IS and related complications.

Clinical and genetic biomarkers are increasingly being used to diagnose and predict disease in stroke patients (38, 39). Li et al. showed that the discharge from hospital of stroke patients suffering from infectious and inflammatory diseases could be accurately predicted through the use of clinical signs and serum biomarkers (18). Another model by Yuan showed that a nomogram composed of diabetes mellitus, hypertension, stroke origins, smoking status, and educational years could be used to predict the risk of recurrent stroke in young patients (40). Bi et al. also developed a risk prediction algorithm for acute ischemic stroke (AIS) for patients presenting with acute dizziness, vertigo, or imbalance in the emergency department (16).

The main strength of this case-control prediction study was the enrollment of people with specific genetic variations. Furthermore, the predictors used in our nomogram are well defined and widely available. The high AUCs achieved by the prediction model for both genders indicate that the risk assessment tool could be used to guide clinical practice. However, there are limitations to this research. The data for this study was obtained retrospectively from a single center. This could limit the generalizability of the research findings due to biases introduced during the self-reported socioeconomic status and testing variations. Furthermore, although the predictive models generated by LASSO are more stable when compared with logistic regression, the restricted number of features identified by LASSO remains a flaw for both male and female models. As a result, the LASSO regression results can only be used as a guide. To overcome this problem, we combined the results of the multivariate logistic regression and univariate regression and incorporated the well-known clinical stroke risk factors to create a predictive nomogram model. Finally, the number of stroke occurrences associated with each predictor was small, potentially limiting the clinical practicability of the model. Consequently, the generalizability of the model needs to be validated using a larger dataset from various centers before it can be used in clinical practice.

## Conclusion

The pri-let-7f-2 rs17276588 polymorphism is strongly correlated with the risk of IS in the northern Chinese Han population. Our developed nomogram, combining genetic signatures and clinical data, offers a lot of potential for predicting the risk of IS in patients. Given the rising socioeconomic costs of IS-related disability and death, methods that make it easier to monitor high-risk groups may reduce the incidence and morbidity of the disease (41–43). If future validation is done, this nomogram could have a lot of potential for assisting in the identification of high-risk groups and primary stroke prevention.

## Data availability statement

The original contributions presented in the study are included in the article/Supplementary material, further inquiries can be directed to the corresponding author.

## Ethics statement

Written informed consent was obtained from the individual(s) for the publication of any potentially identifiable images or data included in this article.

## Author contributions

YXW designed the overall study. YXW, XL, and LC designed and carried out experiments. LQ, YW, and ZH collected the data. YZW wrote the manuscript. YXW and YZW designed experiments, supervised this study, and edited the article. All authors contributed to the article and approved the submitted version.

## Footnotes

1
http://genome.ucsc.edu/

2
https://www.ncbi.nlm.nih.gov/snp/

3
https://manticore.niehs.nih.gov/snpinfo/snpfunc.html

4
https://www.R-project.org

5
http://rna.tbi.univie.ac.at

## References

[1] Sandset EC Goldstein LB. Advances in stroke: treatments-preventive. *Stroke.* (2022) 53:608–10. 10.1161/STROKEAHA.121.036977 34983247

[2] Owolabi MO Thrift AG Mahal A Ishida M Martins S Johnson WD Primary stroke prevention worldwide: translating evidence into action. *Lancet Public Health.* (2022) 7:e74–85. 10.1016/S2468-2667(21)00230-9 34756176 PMC8727355

[3] Ma Q Li R Wang L Yin P Wang Y Yan C Temporal trend and attributable risk factors of stroke burden in China, 1990-2019: an analysis for the global burden of disease study 2019. *Lancet Public Health.* (2021) 6:e897–906. 10.1016/S2468-2667(21)00228-0 34838196 PMC9047702

[4] Wu S Wu B Liu M Chen Z Wang W Anderson CS Stroke in China: advances and challenges in epidemiology, prevention, and management. *Lancet Neurol.* (2019) 18:394–405. 10.1016/S1474-4422(18)30500-3 30878104

[5] Herpich F Rincon F. Management of acute ischemic stroke. *Crit Care Med.* (2020) 48:1654–63. 10.1097/CCM.0000000000004597 32947473 PMC7540624

[6] Qin H Chen Y Liu G Turnbull I Zhang R Li Z Management characteristics and prognosis after stroke in China: findings from a large nationwide stroke registry. *Stroke Vasc Neurol.* (2021) 6:1–9. 10.1136/svn-2020-000340 32571805 PMC8005905

[7] Lu X Niu X Shen C Liu F Liu Z Huang K Development and validation of a polygenic risk score for stroke in the Chinese population. *Neurology.* (2021) 97:e619–28. 10.1212/WNL.0000000000012263 34031205 PMC8424497

[8] Wang Y-Z Zhang H-Y Liu F Li L Deng S-M He Z-Y. Association between PPARG genetic polymorphisms and ischemic stroke risk in a northern Chinese Han population: a case-control study. *Neural Regen Res.* (2019) 14:1986–93. 10.4103/1673-5374.259621 31290457 PMC6676861

[9] Wang Y-Y Zhang H-Y Jiang W-J Liu F Li L Deng S-M Genetic polymorphisms in pri-let-7a-2 are associated with ischemic stroke risk in a Chinese Han population from Liaoning, China: a case-control study. *Neural Regen Res.* (2021) 16:1302–7. 10.4103/1673-5374.301019 33318409 PMC8284288

[10] Wang Y Yin X Li L Deng S He Z. Association of apolipoprotein C3 genetic polymorphisms with the risk of ischemic stroke in the Northern Chinese Han population. *PLoS One.* (2016) 11:e0163910. 10.1371/journal.pone.0163910 27690381 PMC5045204

[11] Mohr AM Mott JL. Overview of microRNA biology. *Semin Liver Dis.* (2015) 35:3–11. 10.1055/s-0034-1397344 25632930 PMC4797991

[12] Rehfeld F Rohde AM Nguyen DTT Wulczyn FG. Lin28 and let-7: ancient milestones on the road from pluripotency to neurogenesis. *Cell Tissue Res.* (2015) 359:145–60. 10.1007/s00441-014-1872-2 24825413

[13] Selvamani A Sathyan P Miranda RC Sohrabji F. An antagomir to microRNA Let7f promotes neuroprotection in an ischemic stroke model. *PLoS One.* (2012) 7:e32662. 10.1371/journal.pone.0032662 22393433 PMC3290559

[14] Yuan F Xiao X Che G Wang Y Wang T Luo X A functional variant in the flanking region of pri-let-7f contributes to colorectal cancer risk in a Chinese population. *J Cell Physiol.* (2019). [Epub ahead of print]. 10.1002/jcp.28227 30740676

[15] Yan Y-X Wu L-J Zhang J Wang S Wang W Dong J Let-7 related genetic variation and risk of metabolic syndrome in a Chinese population. *Endocr J.* (2015) 62:887–96. 10.1507/endocrj.EJ15-0236 26178671

[16] Bi Y Cao FA. Dynamic nomogram to predict the risk of stroke in emergency department patients with acute dizziness. *Front Neurol.* (2022) 13:839042. 10.3389/fneur.2022.839042 35250839 PMC8896851

[17] Wang N Liu H Tian M Liang J Sun W Zhang L A nomogram that includes neutrophils and high-density lipoprotein cholesterol can predict the prognosis of acute ischaemic stroke. *Front Neurol.* (2022) 13:827279. 10.3389/fneur.2022.827279 35280284 PMC8914087

[18] Li J Huang J Pang T Chen Z Li J Wu L Risk estimation of infectious and inflammatory disorders in hospitalized patients with acute ischemic stroke using clinical-lab nomogram. *Front Neurol.* (2021) 12:710144. 10.3389/fneur.2021.710144 34956037 PMC8702498

[19] Friedman J Hastie T Tibshirani R. Regularization paths for generalized linear models via coordinate descent. *J Stat Softw.* (2010) 33:1–22. 20808728 PMC2929880

[20] Sauerbrei W Royston P Binder H. Selection of important variables and determination of functional form for continuous predictors in multivariable model building. *Stat Med.* (2007) 26:5512–28. 10.1002/sim.3148 18058845

[21] Xing J Min L Zhu S Zhang H Zhao Y Li H Factors associated with gastric adenocarcinoma and dysplasia in patients with chronic gastritis: a population-based study. *Chin J Cancer Res.* (2017) 29:341–50. 10.21147/j.issn.1000-9604.2017.04.07 28947866 PMC5592822

[22] Iasonos A Schrag D Raj GV Panageas KS. How to build and interpret a nomogram for cancer prognosis. *J Clin Oncol.* (2008) 26:1364–70. 10.1200/JCO.2007.12.9791 18323559

[23] Balachandran VP Gonen M Smith JJ DeMatteo RP. Nomograms in oncology: more than meets the eye. *Lancet Oncol.* (2015) 16:e173–80. 10.1016/S1470-2045(14)71116-7 25846097 PMC4465353

[24] Pencina MJ D’Agostino RB. Overall C as a measure of discrimination in survival analysis: model specific population value and confidence interval estimation. *Stat Med.* (2004) 23:2109–23. 10.1002/sim.1802 15211606

[25] Vickers AJ Cronin AM Elkin EB Gonen M. Extensions to decision curve analysis, a novel method for evaluating diagnostic tests, prediction models and molecular markers. *BMC Med Inform Decis Mak.* (2008) 8:53. 10.1186/1472-6947-8-53 19036144 PMC2611975

[26] Huang Y-Q Liang C-H He L Tian J Liang C-S Chen X Development and validation of a radiomics nomogram for preoperative prediction of lymph node metastasis in colorectal cancer. *J Clin Oncol.* (2016) 34:2157–64. 10.1200/JCO.2015.65.9128 27138577

[27] Kramer AA Zimmerman JE. Assessing the calibration of mortality benchmarks in critical care: The Hosmer-Lemeshow test revisited. *Crit Care Med.* (2007) 35:2052–6. 10.1097/01.CCM.0000275267.64078.B0 17568333

[28] Wang C Liu S Li J Cheng Y Wang Z Feng T Biological functions of let-7e-5p in promoting the differentiation of MC3T3-E1 cells. *Front Cell Dev Biol.* (2021) 9:671170. 10.3389/fcell.2021.671170 34568312 PMC8455882

[29] Yao Q He Y-L Wang N Dong S-S Tu He Ta Mi Shi ME Feng X Identification of potential genomic alterations and the circRNA-miRNA-mRNA regulatory network in primary and recurrent synovial sarcomas. *Front Mol Biosci.* (2021) 8:707151. 10.3389/fmolb.2021.707151 34485383 PMC8414803

[30] Shin KM Jung DK Hong MJ Kang HJ Lee WK Yoo SS The pri-let-7a-2 rs1143770C>T is associated with prognosis of surgically resected non-small cell lung cancer. *Gene.* (2016) 577:148–52. 10.1016/j.gene.2015.11.036 26625972

[31] Qian Y Chopp M Chen J. Emerging role of microRNAs in ischemic stroke with comorbidities. *Exp Neurol.* (2020) 331:113382. 10.1016/j.expneurol.2020.113382 32561412

[32] Cornier M-A Dabelea D Hernandez TL Lindstrom RC Steig AJ Stob NR The metabolic syndrome. *Endocr Rev.* (2008) 29:777–822. 10.1210/er.2008-0024 18971485 PMC5393149

[33] Reeves MJ Bushnell CD Howard G Gargano JW Duncan PW Lynch G Sex differences in stroke: epidemiology, clinical presentation, medical care, and outcomes. *Lancet Neurol.* (2008) 7:915–26. 10.1016/S1474-4422(08)70193-5 18722812 PMC2665267

[34] Vyas MV Silver FL Austin PC Yu AYX Pequeno P Fang J Stroke incidence by sex across the lifespan. *Stroke.* (2021) 52:447–51. 10.1161/STROKEAHA.120.032898 33493057

[35] Lu Y-W Chang C-C Chou R-H Tsai Y-L Liu L-K Chen L-K Gender difference in the association between TyG index and subclinical atherosclerosis: results from the I-lan longitudinal aging study. *Cardiovasc Diabetol.* (2021) 20:206. 10.1186/s12933-021-01391-7 PMC8515653 34645432

[36] Cui H Liu Q Wu Y Cao L. Cumulative triglyceride-glucose index is a risk for CVD: a prospective cohort study. *Cardiovasc Diabetol.* (2022) 21:22. 10.1186/s12933-022-01456-1 35144621 PMC8830002

[37] Alizargar J Bai C-H Hsieh N-C Wu S-FV. Use of the triglyceride-glucose index (TyG) in cardiovascular disease patients. *Cardiovasc Diabetol.* (2020) 19:8. 10.1186/s12933-019-0982-2 31941513 PMC6963998

[38] Qi J Zhang J Ge X Wang X Xu L Liu N The addition of peripheral blood inflammatory indexes to nomogram improves the predictive accuracy of survival in limited-stage small cell lung cancer patients. *Front Oncol.* (2021) 11:713014. 10.3389/fonc.2021.713014 34692490 PMC8531548

[39] Zhang L Pan J Wang Z Yang C Huang J. Construction of a microRNA-based nomogram for prediction of lung metastasis in breast cancer patients. *Front Genet.* (2020) 11:580138. 10.3389/fgene.2020.580138 33679865 PMC7933652

[40] Yuan K Chen J Xu P Zhang X Gong X Wu M Nomogram for predicting stroke recurrence among young adults. *Stroke.* (2020) 51:1865–7. 10.1161/STROKEAHA.120.029740 32390546

[41] Schwamm LH. In stroke, when is a good outcome good enough? *N Engl J Med.* (2022) 386:1359–61. 10.1056/NEJMe2201330 35388672

[42] Bicciato G Arnold M Gebhardt A Katan M. Precision medicine in secondary prevention of ischemic stroke: how may blood-based biomarkers help in clinical routine? An expert opinion. *Curr Opin Neurol.* (2022) 35:45–54. 10.1097/WCO.0000000000001011 34839341

[43] Feigin VL. Primary stroke prevention: useful thresholds? *Lancet Neurol.* (2022) 21:116. 10.1016/S1474-4422(21)00458-0 34922643

