# Supplementary material for: Genetic variation within the pri-let-7f-2 in the X chromosome predicting stroke risk in a Chinese Han population from Liaoning, China: From a case-control study to a new predictive nomogram
Source: Front Med (Lausanne). 2022 Nov 30;9:936249. doi: 10.3389/fmed.2022.936249 (PMC9747750; doi:10.3389/fmed.2022.936249)
Supplement: Supplementary file 1 [file Table_1.DOCX]

Supplementary Material

**TABLE S1A** | Univariate analysis for the IS risk factors among the male population.

| **Variable** | **Univariate Logistic regression**  **OR (95%CI)** | ***P*-value** |
| --- | --- | --- |
| Age (≤60/>60) | 1.273 (1.052-1.541) | 0.013 |
| BMI (≤22.9/>22.9) | 1.241 (1.032-1.493) | 0.022 |
| Diabetes mellitus | 2.153 (1.709-2.712) | <0.001 |
| Hypertension | 2.908 (2.405-3.515) | <0.001 |
| History of smoking | 1.747 (1.437-2.124) | <0.001 |
| History of alcohol use | 1.769 (1.366-2.290) | <0.001 |
| Hyperlipidemia | 1.460 (1.214-1.756) | <0.001 |
| rs17276588 AA vs GG | 5.032 (4.024-6.292) | <0.001 |

*OR, odds ratio; CI, confidence interval. BMI, body mass index.*

**ORs and 95% CIs were calculated by logistic regression.*

**TABLE S1B |** Univariate analysis for the IS risk factors among the female population.

| **Variable** | **Univariate Logistic regression**  **OR (95%CI)** | ***P-*value** |
| --- | --- | --- |
| Age (≤60/>60) | 1.003 (0.809-1.242) | 0.981 |
| BMI (≤22.9/>22.9) | 1.308 (1.058-1.616) | 0.013 |
| Diabetes mellitus | 1.291 (1.008-1.654) | 0.043 |
| Hypertension | 1.663 (1.345-2.056) | <0.001 |
| History of smoking | 1.706 (1.366-2.130) | <0.001 |
| History of alcohol use | 1.260 (0.960-1.653) | 0.096 |
| Hyperlipidemia | 0.986 (0.794-1.224) | 0.986 |
| rs17276588 AG vs GG | 3.957 (3.102-5.048) | <0.001 |
| rs17276588 AA vs GG | 4.759 (2.724-8.313) | <0.001 |

*OR, odds ratio; CI, confidence interval. BMI, body mass index.*

**ORs and 95% CIs were calculated by logistic regression.*

**TABLE S2A |** Clinical and demographic characteristics of the male patients in the training and validation cohorts.

| **Characteristics** | **Training cohort**  ***(n=1394)*** | **Validation cohort**  ***(n=464)*** | ***P*-value** |
| --- | --- | --- | --- |
| Age (≤60/>60) | 498 (35.7) | 185 (39.9) | 0.121 |
| BMI (≤22.9/>22.9) | 607 (43.5) | 224 (48.3) | 0.085 |
| Diabetes mellitus, n (%) | 335 (24.0) | 91 (19.6) | 0.058 |
| Hypertension, n (%) | 763 (54.7) | 256 (55.2) | 0.912 |
| History of smoking, n (%) | 479 (34.4) | 179 (38.6) | 0.112 |
| History of alcohol use, n (%) | 226 (16.2) | 79 (17.0) | 0.736 |
| Hyperlipidemia, n (%) | 668 (47.9) | 198 (42.7) | 0.056 |

*BMI, body mass index.*

**TABLE S2B |** Clinical and demographic characteristics of the female patients in the training and validation cohorts.

| **Characteristics** | **Training cohort**  ***(n=1051)*** | **Validation cohort**  ***(n=350)*** | ***P*-value** |
| --- | --- | --- | --- |
| Age (≤60/>60) | 435 (41.4) | 137 (39.1) | 0.498 |
| BMI (≤22.9/>22.9) | 486 (46.2) | 159 (45.4) | 0.840 |
| Diabetes mellitus, n (%) | 249 (23.7) | 94 (26.9) | 0.262 |
| Hypertension, n (%) | 545 (51.9) | 195 (55.7) | 0.234 |
| History of smoking, n (%) | 379 (36.1) | 138 (39.4) | 0.286 |
| History of alcohol use, n (%) | 186 (17.7) | 79 (22.6) | 0.053 |
| Hyperlipidemia, n (%) | 414 (39.4) | 126 (36.0) | 0.287 |

*BMI, body mass index.*

**TABLE S3 |** LASSO regression analysis for the IS risk factors.

| **Intercept and variables** | **Regression coefficient** | |
| --- | --- | --- |
|  | **Male** | **Female** |
| Intercept | -1.333 | -0.609 |
| Age (≤60/>60) | 0.112 | . |
| BMI (≤22.9/>22.9) | 0.276 | 0.189 |
| Diabetes mellitus | 0.631 | 0.128 |
| Hypertension | 0.747 | 0.364 |
| History of smoking | 0.242 | . |
| History of alcohol use | 0.248 | 0.930 |
| Hyperlipidemia | 0.497 | . |
| rs17276588 | 0.760 | 1.181 |

*BMI, body mass index.*
